# Oncolytic Virus Therapies in Malignant Gliomas: Advances and Clinical Trials

**DOI:** 10.3390/cancers17193180

**Published:** 2025-09-30

**Authors:** Rin Yang, Jack Hedberg, Jordan Montagano, Malik Seals, Sushant Puri

**Affiliations:** 1 Department of Biomedical Engineering, Oregon Health & Science University, Portland, OR 97201, USA; 2 School of Medicine, Oregon Health & Science University, Portland, OR 97201, USA; 3 Medical Scientist Training Program, The Ohio State University, Columbus, OH 43210, USA; 4 Department of Neurosurgery, Oregon Health & Science University, Portland, OR 97201, USA; 5 Department of Neurology, Johns Hopkins Hospital, Baltimore, MD 21287, USA

**Keywords:** cancer, brain, glioma, immunotherapy, oncolytic virus, herpes simplex virus, adenovirus, poliovirus, neuroimmunology, immune micro-environment

## Abstract

The overall survival rate of brain malignancies such as glioblastoma is currently a little under two years, at most, and treatment options for malignant brain tumors have demonstrated limited efficacy. The current standard of care to treat brain cancer includes surgical resection, radiation, and chemotherapy. Historically, an observed interaction between malignancies and concurrent viral infection has shown therapeutic potential that can perhaps be better leveraged in brain cancer with the technological advances that we have today. We aim to discuss a variety of viral vector designs to harness their oncolytic potential and explore how some of these ideas have performed in clinical trials. In our review, three major viral candidates that have gained traction in this field of research—Herpes simplex virus-1, adenovirus, and poliovirus—are highlighted. How the field has manipulated aspects of their virology and combined these viral platforms with other immune modulating strategies to treat both adult and pediatric tumors is also surveyed. Finally, the work exploring the possibility of other neurotropic viral candidates has been elaborated. More insight into the biological interactions between tumor, brain, and body is needed to address this particularly difficult clinical challenge. While there is still no clear, effective treatment for brain malignancies, the utilization of oncolytic viruses shows potential both as a treatment and as a tool to better understand the immune microenvironment of this pathology.

## Simple Summary

The current prognosis for primary brain cancers such as glioblastoma remains poor despite aggressive clinical measures. Immunotherapy has demonstrated promise in the treatment of many other types of cancers, but this has not been apparent when it comes to brain malignancies. This has necessitated exploring other immune modulating methodologies, one of which has been the utilization of cancer-targeted viruses, also known as oncolytic viruses. In this review, we summarize the progress this field has made in both the preclinical space, which interrogates the still lesser-known biology of brain tumors, as well as the clinical space, through a number of trials that give insight into the therapeutic applicability of a particle that is otherwise a pathogen. By doing so, we hope to provide insight into future directions for expanded treatment options for this patient population.

## 1. Introduction

Primary brain malignancies, especially high-grade gliomas, are of utmost concern due to their poor treatment response and high mortality rates. Each year, roughly 85,000 primary brain cancer diagnoses are made in the United States, of which 29% are malignant. In the malignant cohort, approximately 80–85% are gliomas [1]. Glioblastoma (GBM), a grade 4 glioma, is an especially concerning diagnosis because of its poor overall survival (OS) statistics. Current treatment modalities for glioblastoma include surgical resection followed by concurrent radiotherapy and chemotherapy with temozolomide. Considering the approved therapies and ongoing treatment research, the median OS for glioblastoma ranges from 14.6 to 20.5 months [2]. In the face of ongoing treatment challenges, it remains crucial to continue to look for therapeutics that may offer new insights and improve treatment outcomes in these patients.

Several physiological characteristics of the brain pose unique barriers to the treatment of brain cancers. Among them is the blood–brain barrier (BBB), the highly regulated epithelial layer separating the vascular lumen of blood vessels and the brain parenchyma. The BBB enforces strict regulation of substances transiting to and from the central nervous system (CNS), and, in this process, hinders many cytotoxic chemotherapy candidates from ever gaining entry to the desired target sites [3]. In the context of GBM, the well-established heterogeneity of these tumors, tumor microenvironment (TME), and embedded glioma stem cells (GSCs) further complicates treatment [3,4]. Additionally, while immunotherapies such as checkpoint inhibitors and CAR-T cell therapy have greatly improved outcomes for a variety of cancers, the same dramatic efficacy has not been noted for brain cancers. This is partly ascribed to the fact that tumors in the brain tend to be immunologically “cold”—with poor infiltration of T-cells and numerous mechanisms of immunosuppression unfolding simultaneously in the TME [5]. Despite the formidable barriers to brain tumor therapy that have largely stymied improvements in outcomes for two decades, emerging branches of immunotherapy offer unique strategies for circumventing and even exploiting these brain-unique characteristics to achieve therapeutic efficacy. Here, we survey the landscape of both preclinical and clinical data for such burgeoning brain-tumor-relevant immunotherapies for GBM as well as other kinds of primary brain malignancies. We specifically summarize the work in oncolytic virotherapy and outline how this treatment class holds the potential to transform brain tumor therapy in the 21st century.

## 2. Background

Dating back to the middle of the nineteenth century, case reports of viral infections promoting tumor regression have been noted. More specifically, reports highlighted how different hematological malignancies had marked but temporary beneficial responses in the setting of acute viral infection. These observations served as a precursor to decades of focus and research on oncolytic virus (OV) therapy for many forms of cancer [6]. Advances in molecular virology leading to genetic modifications have made otherwise lethal pathogens available for preclinical research and clinical use. Currently, there is just one FDA-approved OV therapy. This OV, known as T-VEC (commercially named IMLYGIC), is a genetically engineered Herpes simplex 1 (HSV-1) strain (JS1/34.5) indicated for melanoma patients with non-resectable skin and lymph node lesions amenable to therapeutic injection [7].

### Mechanism of Action

While there is much potential for OV therapy, there are some important considerations to be made when utilizing viruses for therapeutic purposes. OVs should utilize the inherent abilities of the infectious agent to attack cancer. An intrinsic feature of viruses is their function as obligate intracellular parasites [8]. Harnessing this function to target malignant cells offers a unique approach to cancer therapy. Such research is being performed on a multi-faceted scale, with respect to modifying OVs for maximal tumor response and combining OVs with other treatment modalities like immune checkpoint inhibitors, targeted drugs, and chemotherapies [9]. Alongside the possible benefits of OV therapy, it is also prudent to consider the risks. Such factors include viral persistence within the host, poor treatment response, ineffective therapeutic administration, and other possible immune sequela arising from an incomplete understanding of how OVs interact with the immune system, cancer cells, and the tumor microenvironment (TME) [10]. As such, careful anticipation and accounting for these possible risks have been cornerstones of modern OV clinical trial design. Much consideration is given to the genomic stability of trial OVs as well as their dosage, timing, and the availability of antiviral medications as an independent means of shutting down viral replication should the need arise.

**Figure 1:**
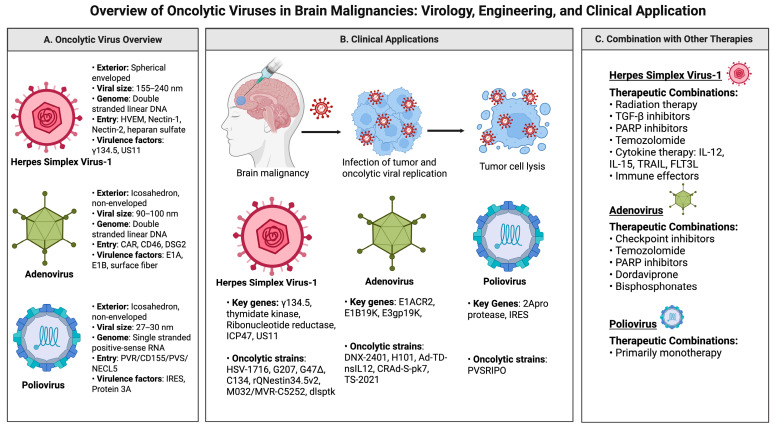
An overview of oncolytic viruses in brain malignancies: virology, engineering, and clinical applications.

Beyond consideration for the risks of OV therapy itself, specified cancer targeting is another crucial aspect of OV design. Cancer cells harbor OV-targetable features including irregularities in cell signaling, metabolism, and homeostasis that may enable OV-therapy-unique tumor selective capabilities that can spare healthy cells [11]. Moreover, viruses can provoke immunogenic forms of tumor cell death via innate and adaptive immune mechanisms including induction of innate antiviral type 1 interferon (IFNα, IFNβ) responses within the tumor, stimulating pro-inflammatory signals in tumor stroma and subsequent recruitment adaptive immune responses. Following tumor cell lysis by OVs, released pathogen-associated molecular patterns (PAMPs) and damage-associated molecular patterns (DAMPs) can aid in stimulating an innate immune response against cancer cells [11]. With regard to brain malignancies, confinement to the brain and the surrounding tissue largely constituted of post-mitotic cells provides a powerful opportunity for viral therapy. This is because treatment can be focused on one well-compartmentalized organ with less concern for off-target systemic side effects. Additionally, in order to replicate, many viral candidates require active cell division, which again provides a method for tumor targeting and the preservation of neighboring healthy non-cancerous cells [12]. Following the footsteps of T-VEC, the field of neuro-onco-immunology has seen progress with oncolytic HSV-1 strategies along other viral vectors surveyed in the following section. Figure 1 summarizes the virology, molecular engineering, and combination treatments discussed in this article.

## 3. Herpes Simplex Virus-1

Herpes simplex virus-1 (HSV-1) is an enveloped, double-stranded DNA virus with a 152 kb genome containing approximately 84 genes. Multiple host-cell entry receptors, namely nectin-1, nectin-2, Herpes virus entry mediator (HVEM), and 3-O-sulfated heparan sulfate, are exploited by HSV-1, and this allows for neural and surface epithelial tropism of this virus. Wild-type versions of HSV-1 are highly neurovirulent, primarily due to the γ_1_34.5 gene, which possesses multiple functions including reversal of host-cell translational shutoff that is otherwise triggered by cytosolic double-stranded RNA produced by HSV-1 [13,14,15]. Most oncolytic HSV-1 (oHSV) vectors are attenuated by the inactivation or deletion of this virulence factor and by disabling other viral genes that are essential for viral replication in normal cells but not tumor cells. An example of such a gene is viral ribonucleotide reductase, which is often functionally disabled by insertion of lacZ from *Escherichia coli*. However, these additional manipulations tend to reduce oncolytic activity [16].

HSV-1 possesses several unique qualities that have facilitated its advancement into oHSV. It is one of the most studied viruses, with a large genome that can accommodate lengthy inserts, enabling the development of modified oHSVs whose added gene products impart novel functions that may then be assessed for anti-tumor efficacy and safety. Furthermore, the viral DNA does not integrate into host genomes, reducing the likelihood of insults to host genome integrity caused by the virus. Finally, multiple classes of antiviral therapies exist that are effective against HSV, providing a safety net in which viral activity can be halted if necessary during the course of clinical trials. The virology, translational science, and clinical performance of oncolytic HSVs in both brain malignancies as well as the broader array of cancers have been well described [17,18,19,20,21,22,23,24,25,26,27].

### 3.1. Preclinical Findings of oHSV

Advances are being made regarding the more basic understanding of how brain tumor biology, specifically its modulation of the regional immune system, responds to various facets of oHSV activity. It was recently demonstrated that tumor cell MHC II expression increases as a result of oHSV therapy in murine syngeneic models, facilitating CD4+ T cell killing [28]. Further, oHSV therapy has been shown to induce the presentation of tumor antigens, consistent with the idea that oHSVs may be acting as ‘in situ tumor vaccines’ [29]. On the other hand, innate immune cells may limit oHSV efficacy by lysing and clearing oHSV-infected tumor cells before the oHSV has had opportunity to spread extensively or generate an adaptive immune response [30], and certain approaches have attempted to moderate the early influence of innate immune cells. Pre-treatment of malignant gliomas with transforming growth factor beta (TGFβ), an immunosuppressive cytokine perhaps more conventionally known for its tumor-supportive roles, improved efficacy of oHSV therapy [31]. Interestingly, the inhibition of TGFβ signaling via a TGFβ receptor inhibitor treatment also led to improved oHSV efficacy in patient-derived glioma models [32]. Concurrently, it has also been shown that the GBM TME protein cellular communication network factor 1 (CCN1) is an oHSV-inhibitory factor [33].

Progress has also been made in terms of delineating the molecular pathways responsible for host tumor-cell susceptibility to oHSV infection. A recent report demonstrates that oHSV therapy increases formation of neutrophil extracellular traps (NETs) which may form an impediment to therapeutic efficacy. However, this phenomenon can be interrupted via the use of bromodomain and extraterminal (BET) protein inhibitor [34]. The same group also recently demonstrated that expression of the ICP0 genes during oHSV infection leads to suppression of host cell METTL-14, inducing the anti-tumor effects of oHSV therapy in glioma [35]. It has also been shown that mutation in the Isocitrate Dehydrogenase (IDH) gene compromises the antiviral interferon response, and the metabolite that accumulates with IDH mutation, D-2-hydroxyglutarate, improves oHSV replication in glioma cells [36]. Some groups have employed a systems biology approach to map tumor-specific dependencies that facilitate optimal choices of oHSV-based cytokine expression in different tumors [37]. Bhatt et al. used a computational approach to simulate and understand factors critical for the outcome of oncolytic virotherapy, such as growth of tumors, timing of OV administration, and spatial localization of OV-infected tumor cells, identifying that faster spread of OVs and strong immune signals are important for effective anti-tumor responses [38].

Within oHSVs, major effort is underway to develop novel viruses capable of effectively reprogramming the immune TME to orchestrate more robust anti-tumor immune responses. Diverse transgenes that have been introduced into modified oHSV genomes for such purposes include the following gene products: (1) molecules that amplify the activities of T cells and NK cells such as IL-2 [39], IL-12 [40,41,42,43,44,45], IL-15/IL-15 receptor alpha [46,47], IL-18 [44], and IL-27 [48]; (2) molecules that enhance antigen presentation or that enrich the source of tumor antigens via promoting tumor cell apoptosis such as FLT3 ligand [49] and tumor necrosis factor-related apoptosis-inducing ligand (TRAIL) [50], respectively; (3) molecules that induce trafficking of desirable immune effector cells into the TME to facilitate anti-tumor activity including CCL2 [51], CCL5 [52,53], and CXCL4 [54]. Beyond the expression of cytokines, a wide array of tumor- and other immuno-modulatory transgenes have also been tested in oHSVs, creating multimodal therapies stemming from oHSV platforms that demonstrate potential to engage multiple tumor-killing pathways. This includes the expression of antibodies directly from oHSV [55], immune checkpoint inhibitors [56,57], anti-angiogenic factors [58,59,60], E-cadherin [61], Matrix Metalloproteinase 9 [62], and tumor antigens [63]. This rapidly evolving, diverse family of armed oHSVs allows a wide range of immunotherapeutic and tumor-modifying payloads to be delivered through the tropism and expression dynamics of the OV, contributing to increased tumor specificity and even amplification of the respective genetic payloads.

Further significant progress has been made in trialing oHSV therapies in combination with other modalities of cancer therapy. This includes oHSV in combination with chemotherapy and targeted cancer agents that disrupt DNA repair and induce apoptosis, including the alkylating agent temozolomide [64,65] (though this drug demonstrated mixed results, potentially due to chemotherapies harming tumor cells as well as tumor-infiltrating immune cells), topoisomerase II inhibitors [66], and Poly (ADP-ribose) polymerase (PARP) inhibitors [67]. Further combination agents incorporated alongside oHSV therapy include either those that act via interruption of tumor-supportive functions such as MEK inhibitors [68], NOTCH signaling inhibitors [69], integrin-blocking antibodies [70], and vascular endothelial growth factor receptor (VEGFR) inhibitors [71], or, conversely, those that act via the restoration of tumor-suppressor molecules otherwise frequently corrupted by tumorigenesis such as PTENα [72]. Still more combinatorial therapies have been studied, such as epigenetic modifying drugs capable of reversing functionally repressed or silenced gene expression programs and shifting TMEs toward a more immunostimulatory state [73,74,75], proteasome inhibitors that induce endoplasmic reticulum stress in metabolically active cells [76], and tumor antigen peptide vaccines that could harness the immunostimulatory activity of oHSVs to more precisely direct resulting immune responses against tumor antigens [77]. Combinatorial strategies are clearly the focus of a broad array of oHSV studies, which may foreshadow progression into clinical studies that build upon insights from first-generation oHSV trials to apply these therapies in an increasingly sophisticated combinatorial treatment space.

### 3.2. Clinical Trials of oHSV

Significant progress is being made in the manufacturing and clinical testing of oncolytic Herpes viruses for brain tumors. Several major oHSV derivatives are now being tested in a growing number of clinical trials, providing invaluable information about how these unique modified oHSVs, each modified with unique strategies, perform in patients.

The toxicity of the γ_1_34.5 null strain of HSV, HSV-1716, was assessed in patients with recurrent astrocytoma and glioblastoma. When administered by stereotactic injection directly into the tumor, doses up to 10^5^ pfu of HSV1716 were well tolerated with no evidence of systemic shedding in saliva or serum. While the aim of this study was to study the safety profile of this virus, it is notable that, of the nine total patients, four patients showed a response to treatment that ranged between complete remission after further tumor decompression, demonstrative clinical and radiologic stability, and increased survival past 24 months. Histological studies only assessed for encephalitis, which showed that the cause of death in the other five patients of this study was not due to the virus. However, it is unclear if there were differences in immune penetrance to explain the varied responses to virus administration [78,79].

Similarly to HSV1716, G207 is an attenuated oHSV that still has γ_1_34.5 deletion and also has an insertion of the *E*. *coli* lacZ gene to disable viral ribonucleotide reductase, ICP6. As mentioned previously, while these manipulations can increase the safety profile of these viruses, they can reduce oncolytic activity, and this necessitates testing in clinical trials. After assessing its safety profile, Markert et al. tested the efficacy of this virus in combination with radiation. Findings suggest that there was no evidence of adverse events due to uncontrolled viral dissemination, and the median OS was 23 months from initial diagnosis. Histological studies pre- and post- treatment generally showed an increase in tumor infiltrating lymphocytes (specifically CD3+ and CD8+ lymphocytes) and increased innate immune cell populations. In contrast, B cell (CD20+) infiltration was relatively unchanged [79,80,81]. This same virus was evaluated for safety when combined with radiation treatment in a group of 13 pediatric patients with progressive or recurrent high-grade IDH wild-type gliomas. Up to the maximum dose of 10^8^ pfu and 5 Gy of radiation, there were minor adverse events possibly attributed to G207, but there was no viral shedding, or dose-limiting toxic or serious adverse events. Median OS was 12.2 months, and tissue studies after treatment showed an increased level of infiltrating T cells post-treatment compared to pre-treatment [82].

G47Δ was developed in the US using G207 oHSV, but with a third modification: deletion of α47—an HSV gene that interferes with host-cell antigen presentation. This modification additionally results in disruption of the promoter for the US11 gene, endowing it with immediate-early kinetics that help to compensate for γ_1_34.5 deletion and balance retention of the virus’ oncolytic capabilities with attenuation. G47Δ has shown promise as a therapeutic in clinical trials in Japan for GBM and recently became the first oncolytic virus to be clinically approved for this pathology [83,84,85].

Another way to strike the balance of ensuring a good safety profile while maintaining the anti-tumorigenic attributes of oHSV has been to arm γ_1_34.5 deleted oHSV with immunostimulatory molecules. M032 is a γ_1_34.5-deleted IL-12-expressing strain of oHSV that showed an acceptable safety profile in a clinical trial (NCT02062827) in patients with malignant gliomas [86,87]. There is also another clinical trial (NCT05084430) currently recruiting to study the safety and efficacy of M032 and Pembrolizumab (anti-PD-1 antibody) combination therapy. Another example of a similar virus design strategy is MVR-C5252, a γ_1_34.5-deleted IL-12- and anti-PD-1-antibody-expressing strain that is also in clinical trials at the time of this review (NCT06126744). Preliminary findings reveal that MVR-C5252 delivered through convection-enhanced delivery—a route of administration that bypasses the BBB—is well tolerated in patients with high grade gliomas. Assessment of therapeutic efficacy is currently ongoing [88,89].

Two other designs of oHSV also in ongoing clinical trials are rQnestin34.5v2 (NCT03152318) and C134 (NCT03657576). rQnestin34.5v2 is a virus with both copies of the wild-type γ_1_34.5 gene deleted, and with an insertion of a nestin promoter-driven γ_1_34.5 gene. What this allows for is partial restoration of this virulence factor but only in cells, such as GBM cells, that overexpress nestin [90,91]. Developed in 2005, C134 is a chimeric oHSV that again contains the deletion of γ_1_34.5 but also has the additional insertion of the cytomegalovirus IRS1 gene, which increases late viral protein synthesis and is designed to improve upon some of the attenuation otherwise observed in γ134.5-deleted oHSVs without compromising safety [92,93,94].

Beyond oHSVs, a broader array of oncolytic viruses continues to be explored both in brain tumors and for a variety of other tumor types, contributing to an increasingly solid network of foundational knowledge enabling progress in terms of benchmarking crucial factors for design, engineering, clinical administration, and therapeutic monitoring of oncolytic viruses [95,96].

## 4. Adenovirus

Adenoviruses (Ads) are a family of naked, double-stranded DNA viruses with an icosahedral shape containing a 26–45 kb genome [97,98]. There are 51 known serotypes of human Ad, and they are divided into different subgroups, A to F [99,100]. A majority of oncolytic Ads (oAds) infect cells via the cocksackie and adenovirus receptor (CAR) while some serotypes, namely a subset of serotypes B (i.e., Ad16, Ad21, Ad35, Ad50) and D use CD46. Desmoglein 2 (DSG2) is another identified receptor for a different subset of serotype B Ads that include Ad3, Ad7, Ad11, and Ad14, though both Ad11 and, partially, Ad3 interact with both CD46 and DSG2 [101,102]. Once internalized, particles of the pathogen containing the viral genome travel along microtubules to the nucleus where genes E1A and E1B start the cascade of viral gene replication and transcription. The infected cell then enters S-phase as the conserved region (CR) 2 within the E1A protein replaces retinoblastoma (Rb) proteins associated with the E2F transcription factor. Following, proteins encoded by the E1B gene prolong viral replication by preventing cell death post-infection by either directly acting as an anti-apoptotic factor or binding to and subsequently inducing the degradation of p53 [97,98].

There are several advantageous characteristics of Ads that hold promise as OV candidates. The basic virology of Ads is relatively well-described, manufacturing techniques that enable high-titer stock production are well-developed, and the Ad genome can be manipulated with relative ease. Furthermore, Ads and basic components of Ads—the viral capsid, genome, and transcription intermediates—are robust pathogen-associated molecular patterns (PAMPS), which may be leveraged in oAd therapy to induce a pro-inflammatory response in the TME by modulation of cytokines such as interferon (IFN), interleukin-1β (IL-1 β), and IL-18 [100,103]. Furthermore, it has been demonstrated that oAds, like other OVs, also induce an adaptive anti-tumor response by triggering immunogenic cell death. T-cells play a major role in this adaptive response and can be cross-reactive across different serotypes of Ad [98,100].

Although oAds possess significant therapeutic potential, a major barrier to their successful clinical implementation is their diverse tropism, allowing infection of many different healthy tissues in humans. One powerful strategy for overcoming this challenge has emerged from engineering attenuated strains of oAds containing disruptions in viral fitness genes that are dependent on interaction with host cell factors found frequently disrupted in tumors. This way, replication of these attenuated oAds is severely restricted in healthy host cells but persists in tumor cells. For example, the viral gene E1B allows adenovirus evasion of host cell apoptosis by degrading p53, which itself is frequently disrupted in cancers. H101, a modified oAd approved for the treatment of head and neck cancer in China, contains a deletion of E1B, and thus in healthy cells with intact p53, the virus cannot prevent the host cell from triggering apoptosis and thus replication is restricted. However, in tumor cells lacking p53, the virus can efficiently replicate.

Other efforts to improve tumor infiltration of oAds have sought to modify viral capsid surface fibers responsible for directing host-cell entry toward cell-surface receptors frequently enriched in tumors. Specifically, with Ad5, the most commonly used oAd backbone, this constitutes constructing chimeras of the surface fibers between different Ad strains to expand the virus’ cross reactivity. Another approach to modifying oAd surface fibers is incorporating arginine, glycine, and aspartic acid peptides (RGD motifs), and this broadens the spectrum of cellular receptors enriched in gliomas (e.g., integrins such as αvβ3 or αvβ5) [98,100,103]. DNX-2401 (Ad5-Δ24-RGD, Tasadenoturev) is an example of an oAd that has a 24-base pair deletion of a sequence in the conservative region 2 (CR2) domain of the gene E1A and expression of RGD motifs on its surface fibers. As mentioned previously, the CR2 domain binds Rb protein to perpetuate the survival of the infected cell and sustain viral replication. Therefore, with this fiber modification and gene deletion, the design of this virus intends for viral enrichment in gliomas, selective survival in cancer cells with a dysfunctional Rb pathway, and mitigation of its spread in healthy cells. DNX-2401 has shown promise in several clinical trials [98,100,103,104,105].

### 4.1. Preclinical Finding of oAds

Leveraging what is known regarding Ad virology, a wide array of emerging preclinical work contributes to the ongoing momentum of oAds. One of the challenges for OV therapy when it comes to brain malignancies, such as GBM, is that tumor-associated macrophages (TAMs) can phagocytose OVs and limit their therapeutic efficacy by lowering viral titers. Considering the role that TAMs play in the role of tumor progression and relapse, Zhu et al. designed an oAd with E1b-55kD gene deletion with a Ad5 backbone that would also release anti-PD-1, a T cell checkpoint inhibitor, and alendronate, a bisphosphonate drug found to suppress the proliferation and function of TAMs. Expression of these modified elements was programed to be responsive to reactive oxygen species (ROS) within a tumor. With this design they were able to demonstrate increased anti-tumor response across various murine brain tumor models [106].

Beyond the more expected combinations of Ad with CAR T cell or immune check point inhibitors, additional studies investigating oAds in combination with other adoptive cell therapies and pharmacotherapies are underway. γδ T cells expressing the Vγ9Vδ2 TCR have previously demonstrated anti-tumor activity by detecting the increased metabolic stress of tumor cells [107]. αβ T cells engineered with the Vγ9Vδ2 TCR were tested in conjunction with DNX-2401 in in vitro models of pediatric diffuse midline gliomas (DMG). This combination showed an additive cytotoxic effect at relatively low doses of oAds [108]. As for pharmaceutical combinations, when mice with orthotopically implanted brain tumors were administered an oAd, TS-2021 (an oAd with replicative functions under a Ki-67 driven promoter, TGF-β2 5′UTR insertion upstream of the E1A gene for improved tumor specificity, and modifications to constitutively expressed IL-15), it was found that Poly(ADP-ribose) polymerase (PARP) expression increased. Administration of the PARP inhibitor, Olaparib, then demonstrated synergistic effects with this in vivo model. Based on observations of increased γH2AX accumulation after this combination therapy, the investigators of this study speculate that a possible mechanism for oAd-Olaparib synergy could involve oAd-induced DNA damage followed by failure of repair mechanisms in the presence of Olaparib, effectively inducing cell death [109]. Another oAd combination therapy specific for pediatric DMG showing preclinical efficacy is DNX-2401 and dordaviprone, a mitochondrial caseionolytic protease P small molecule inhibitor and dopamine receptor D2 antagonist. Dordaviprone was an attractive drug to test because it was found to have promise when it was given to patients with H3K27 mutant DMG within a 1–3.8-month window after standard-of-care radiation [110]. Using both pediatric high-grade glioma and H3K27M mutant DMG cell lines, the combination of these treatments outperformed the anti-tumor activity of any given single agent both in vitro and in vivo. A possible mechanism of this response was tracked to be an interplay between viral TME reprogramming and dordaviprone-induced DNA damage [111].

Lastly, an emerging field of optimization of oAds is the delivery method of these viral particles. Though the large majority of current OV therapy studies have administered viral particles peritumorally, this is often not an ideal route for administration due to the invasive nature of stereotactic or intracranial procedures, and other routes of administration remain to be explored. In this regard, there are a new set of challenges that include peripheral neutralization of the virus and ensuring both tissue tropism and tumor specificity that require study. As discussed above, one strategy to address some of these issues is to modify the virus so that it better “sees” tumor cells by leveraging the differences between healthy and cancerous cells. However, Wang et al. explore a different strategy by designing a delivery system that encapsulates oAds in a synthesized tannic acid (TA) and ferric ion (Fe^3+^) nanomaterial that could better protect the oAd from peripheral antibody elimination. Their results show that this nanoparticle material is protective against neutralizing antibodies with marked viral load in the tumor after systemic administration. Furthermore, in their in vivo GBM model, the nanoparticles seem to be interacting with the local TME by inducing a more pro-inflammatory state and increasing oAd anti-tumorigenic activity [112]. This perhaps speaks to one of the unique ways oAds and other OVs can be administered for an enhanced therapeutic effect.

### 4.2. Clinical Trials of oAd

Most of the clinical findings with oAds are based on DNX-2401 or a derivative of this strain. As mentioned previously, DNX-2401 is an oAd with a partial deletion of the E1A gene that allows it to be replicated in tumor cells that do not have a functional Rb pathway. Another element of DNX-2401’s design is that its capsid surface fibers are modified to have RGD motifs, which allow it to be enriched in gliomas and allows the virus to escape neutralizing antibodies that jeopardize oAd efficacy [104]. In a cohort of 37 patients with GBM, there was a difference in median OS between the group that received two doses (one prior to surgery and one after resection of the tumor) of the virus (13.0 months) and the group who received one intra-tumoral dose of the virus (9.0 months) with no observed dose-dependent toxicity. In follow up studies, a cohort of patients given a single intra-tumoral dose of DNX-2401 in combination with 200 mg of the PD-L1 checkpoint inhibitor pembrolizumab was found to have a median OS of 12.5 months. When patients were stratified based on the degree of inflammation sampled in the TME, only the “medium” category contained complete responders to this combination therapy, suggesting that a certain ideal level of immune infiltration may play a key role in oAd therapeutic response. Furthermore, DNX-2401 has shown potential applications isn pediatric diffuse pontine intrinsic glioma, an aggressive subtype of glioma with limited treatment options. The median OS when patients of ages 1–18 were given cerebellar infusions of DNX-2401 in two escalating doses (if tolerated; if not, two equivalent doses) was 17.8 months, as compared to the standard-of-care median OS of less than 12 months [98,100,103,105].

oAds more extensively modified than DNX-2401 have also been tested in phase-1 human clinical trials. Ad-TD-nsIL12 is an oAd reported to have favorable outcomes in esophageal squamous cell carcinoma that has recently also been tested to treat recurrent high-grade gliomas. It was designed with a triple deletion of the E1 genes that help establish the infection (specifically E1ACR2, E1B19K, E3gp19K) and insertion of an IL-12 gene lacking its signal peptide sequence. This allows Ad-TD-nsIL12 to express but not secrete IL-12, which would impart a localized immunostimulatory effect of the cytokine in the TME and prevent the toxic effects of systemic IL-12 release [113]. In a dosing phase 1 trial (ChiCTR2000032402) it was found that 1 × 10^10^ pfu could be administered safely intratumorally. Median survival after viral treatment was 5.1 months, but among this cohort of patients there were noted cases of complete and partial response [114]. Another engineered virus in recent clinical trials, CRAd-S-pk7, was designed to have E1A expression under a survivin-driven promoter. Survivin was considered as a tumor targeting strategy because it is highly active in gliomas, as it allows tumor cells to replicate and avoid apoptosis [115]. CRAd-S-pk7 also has a modified Ad5 fiber protein with a polylysine sequence to enhance neural tropism. Following the characterization of its oncolytic potential preclinically, Fares et al. demonstrated the safety profile of CRAd-S-pk7 in a phase 1 dose-escalation trial (NC03072134). In this trial, viruses were loaded in neural stem cells (NSCs) and injected into glioma cavities post-resection, followed by standard-of-care radiation and temozolomide treatment. Up to 1.875×10^11^ viral particles loaded into 150 million NSCs were tolerated without notable side effects. The median progression-free survival and OS were 9.05 months (95% CI 8.54, NA) and 18.4 months (95% CI 15.7, NA), respectively [116].

In summary, oAds such as DNX-2401 and conventional combinations of oAd and immune checkpoint inhibitors have not previously shown the most observable improvement in clinical outcomes. However, these preclinical and clinical findings offer insight into the biology of brain malignancies as well as novel exploratory directions ranging from different drug/virus combinations or altered delivery methods. Notably, the applications of oAds in aggressive pediatric tumors such as DMG and diffuse pontine intrinsic gliomas offer a therapeutic opportunity for this population that currently faces particularly limited options and poor prognoses. This possibly adds value to oAds as a viral candidate that hopefully may provide the field with more insight into these particularly difficult clinical challenges.

## 5. Poliovirus

Throughout history and across the world, there has been abundant documentation of poliomyelitis and the adverse effects of poliovirus infection [117]. Although the wild-type virus has caused widespread disease, more work has sought to transform poliovirus into an OV.

Poliovirus is a naked, icosahedral virus with a positive-sense single-stranded RNA genome. It is transmitted via a fecal–oral route and there are three virus serotypes. A vast majority of infectious poliomyelitis cases are asymptomatic or mild in severity. However, in approximately 0.1–1% of infections, poliovirus infection results in paralysis [118].

While there may be concerns for infection or negative health outcomes, poliovirus has a potential benefit through its anti-cancer activity. The specific host-cell receptor for this virion has multiple aliases, including Cluster Differentiation 155 (CD155), Human Poliovirus Receptor (PVR), Poliovirus Sensitivity Gene (PVS), and Nectin-like Molecule 5 (NECL5/Necl-5) [119]. This receptor is present in many types of tissue, including gastrointestinal and nervous tissue. Interestingly, CD155 has near-universal ectopic expression in solid neoplasms, including GBM [120,121], and rat GBM models have demonstrated that this increased expression may aid in cancer cell invasiveness [122]. Following virion interaction with CD155, viral RNA is inserted into the host cell. The rate limiting step in this process is the translation of the viral RNA. If this step is successful, viral protease 2Apro is the first viral protein released. Within roughly an hour, 2Apro can intercept host-cell genes, which shuts off protein synthesis in the host cell [123].

While this tumor-skewed tropism is beneficial for OV therapy, additional modifications are needed to attenuate the virus prior to human testing. Such attenuation has classically been achieved via genetic modification of the internal ribosome entry site (IRES) of the live attenuated type 1 poliovirus vaccine with the IRES of human rhinovirus type 2 (HRV2) [124]. This nonpathogenic genetically recombinant oncolytic poliovirus is referred to as Polio–Rhinovirus Chimera (PVSRIPO) [121,124,125,126]. Much work has been performed to characterize this nominal strain of poliovirus in both preclinical and clinical spaces.

### 5.1. Preclinical Findings of Poliovirus

Specifically in cancer cells, PVSRIPO can have increased viral translation due to the absence of HRV2 IRES-mediated translation repression [123]. In other words, viral attenuation is limited to healthy cells and not afforded to cancer cells. Additional work has identified that this engineered virus has a unique relationship with the cytosolic pattern recognition receptor (PRR) Mda5, which activates anti-viral/pro-inflammatory responses [127]. Similarly, one intra-tumoral injection of PVSRIPO in syngeneic, immunocompetent rodent tumor models produced systemic anti-tumor cytotoxic responses. This is first marked by neutrophils invading the tumor, which then primes dendritic cells and T cells to access the TME. With antigen-presenting cells present, the sharing of tumor-associated antigens between these cells and cytotoxic T cells allows for the propagation of an adaptive anti-tumor response [128]. Given that there is high uptake of PVSRIPO by tumor cells, assuring viral attenuation is critical for patient safety. It has been shown that serial passages of PVSRIPO in GBM xenografts in vivo models do not generate adapted variants with genetic signatures known to mediate neurovirulence [96]. This work also reveals sustained tumor regression and glioma elimination following PVSRIPO treatment [129].

### 5.2. Clinical Trials of Poliovirus

The extensive pre-clinical studies of oncolytic poliovirus have provided ample evidence supporting advancement in clinical studies, with a key feature being the ability of these viruses to replicate and remain restricted to target tissues [98]. Thus far, there has been one completed clinical trial in adult patients with recurrent GBM treated with intra-tumoral infusion of PVSRIPO by convection-enhanced delivery to overcome the limitations of the BBB. This was a Phase 1 trial, and the primary objective was to determine the toxicity profile and safe dose for Phase 2 studies. From May 2012 to May 2017 a total of 61 participants were enrolled to receive PVSRIPO, and these patients had a median overall survival (OS) of 12.5 months (95% CI 9.9 to 15.2) compared to the historical median OS of 11.3 months (95% CI 9.8 to 12.5). In total, 69% of patients receiving PVSRIPO had a grade 1 or 2 adverse event (AE) attributable to the study therapy. In the dose-expansion phase, more than 20% of patients experienced AEs, with the top four being headache (52%), pyramidal tract syndrome (hemiparesis) (50%), seizure (45%), and cognitive disturbance (25%). A total of 19% of AEs from this phase were at least grade 3 in severity. The study also described preliminary testing of immune-cell frequencies in the periphery suggestive of immunosuppressive regulatory T cell reduction. This suggests enhanced immune-mediated anti-tumor activity in the setting of receiving the first cycle of chemotherapy for tumor progression after PVSRIPO [130]. Based on these findings, PVSRIPO was granted a breakthrough therapy designation by FDA in 2016.

Additionally, PVSRIPO was recently studied in a Phase 1 clinical trial in recurrent pediatric high-grade glioma. The median age of trial patients was 16.5, with a range of 9–19 (n = 8). Patients received a single dose of PVSRIPO via convection-enhanced delivery with the dosage being 5 × 10^7^ pfu, which was 50% of an in vitro tested infectious dose. At the time of reporting, the median OS for this study was 4.13 months, with one patient being alive for more than 21 months. A total of 26 treatment-related AEs were documented in six patients, with none of them meeting grade 4 or 5 criteria [131].

## 6. Other Viruses

Though here we have primarily emphasized efforts to develop oncolytic Herpes viruses, adenoviruses, and polioviruses as brain tumor therapies, there are additional studies that seek to evaluate other viruses based on their intrinsic characteristics such as tissue tropisms or manipulability. Zika virus (ZIKV), despite its aggressive infectious profile, has been studied for its neurotropism. Recently, Ferreira et al. have developed a model of tumor cells co-cultured with cerebral organoids that allows for study of tumor growth and development. Using this model, it was found that when tumoroids generated with pediatric medulloblastoma and atypical teratoid rhabdoid tumor (ATRT) cell lines were infected with ZIKV, there was a larger accumulation of viral RNA compared to organoids made without these tumor cell lines, suggesting a greater replication rate of the viruses in those cells that could potentially be leveraged for therapeutic purposes [132]. Mechanisms that drive neurotropism, especially with different administration routes of viruses, also elucidate Semliki Forest Virus as a potential new neurotropic viral vector. Neuroinvasion of this virus when administered intravenously has been found to be dependent on VLDLR which is expressed on the choroid plexus cells of the brain [133].

Neurotropic viruses that are not pathogenic to humans have also become interesting targets to explore as OV candidates. Vesicular stomatitis virus (VSV) is a part of the Rhabdoviridae family, also known for its tropism for neural tissue, and has demonstrated oncolytic potential due to its rapid induction of apoptosis in infected cells. It markedly has been explored for its synergy with a minimally invasive treatment modality called photodynamic therapy, in which a photosensitive compound such as porphyrin is administered in conjunction with irradiation treatment from a light source [134]. Moreover, novel delivery methods with these less explored viruses have allowed for the potentially enhanced crossing of the BBB, increasing penetration into tissue of the CNS. Myxoma virus, another nonpathogenic virus that lacks the anti-apoptotic M011L gene, loaded into adipose-derived mesenchymal stem cells was found to better penetrate the BBB and survive systemic immunity in both in vitro and murine in vivo models compared to the naked virus alone [135]. Table 1 lists the current trials with oncolytic viruses, including virus type, viral backbone, and the trial phase.

## 7. Discussion

In combination, the field of oV therapy in brain malignancies holds a lot of potential. Both the expansion of utilizing previously explored viral platforms (HSV-1, Ad, and poliovirus) and the work performed on harnessing new viral candidates offer a plethora of new directions to address this particular clinical challenge. The recent discoveries regarding OV therapy strategies can be grouped into the following main ideas: boosting the anti-tumor cytotoxicity of T cells, suppressing innate immunosuppressive cells in the TME, and improving the delivery and design of viral therapies for better tissue targeting and treatment potentiation.

When it comes to boosting the cytotoxicity of T cells for their anti-tumor activity, there are a few trends across the different viruses to note. As a single agent treatment modality in clinical trials, OVs seem to not show dramatic progress in invigorating the immune system for anti-tumorigenic activity. This could possibly be because the TME is already an immune cold environment and attempting to revitalize the scant immune landscape is not very effective. This could also explain why more traditional combination therapies such as ones with check point blockade have not been as successful with brain cancers as they have been with other cancers. The studies above seem to have shifted combining OVs with drugs that target DNA repair mechanisms such as PARP inhibitors. Preclinically, preventing DNA repair and reducing genomic stability seems to bolster immune-driven apoptosis, and this could perhaps be a more viable strategy that better utilizes the existing immune players in the TME of brain tumors.

To address an opposing mechanism, instead of ramping up the immune system, suppressing its inhibitory functions in alternative ways to the immune checkpoint pathway has been an approach that also shows promise. However, it seems that understanding exactly what inhibitory signals to block in the TME and the therapeutic effectiveness of these remains unclear. For example, of the studies mentioned in this discussion, it is not clear whether the role of a cytokine like TGFβ that normally plays an immunosuppressive role in other cancers has a different role in the brain, or how this speaks to its potential as a target for this disinhibition strategy.

Lastly, a large portion of these recent studies have been focused on modulating the viral vector to better target tumors and increase its tropism for the tissue in which the tumor resides. These include modifications such as the introduction of transgenes or arming viruses with different surface receptors that are enriched in neural tumors or tissues. Alternatively, there seems to be some potential in loading viruses up in different cellular vehicles or coating them with nanoparticle technology that can better deliver viruses to the tumor than general systemic administration and therefore potentiating any effects of OVs using higher targeted doses in the tumor.

Overall, it remains unclear how different facets of the brain’s biology interact with the rest of the body’s biology. This is to say, much of what has been explored has been limited by the lack of understanding of how the brain’s immune system communicates with systemic immunity or understanding the extent to which the brain’s physiology is compartmentalized from the rest of the body. Though there has been significant effort in finding therapeutics for brain cancers, there has been a lag behind other cancers that have found benefits in options such as immune check point blockade or OV-immune check point blockade combination therapy. This perhaps can be attributed to the lack of models that can recapitulate brain tumor development, the difficulty of accessing brain tissue due to the invasiveness of procedures that would allow such access, and knowledge gaps in brain immunology and physiology. Especially in the last decade, assumptions about the brain’s immune-privileged state and the ability of cellular immune players to cross the BBB have found new perspectives, and this has pushed novel ideas and approaches for therapeutics. This knowledge gap is even more evident when it comes to the field of oncolytic viruses and when the rationale of viral designs does not seem to translate to mechanistic expectations in vitro while they seem to demonstrate potential in vivo and in clinical trials. For example, it was recently demonstrated that increased levels of oAd receptors (e.g., CAR, DSG2, CD46, and integrins αvβ3 or αvβ5) in tumor cells did not correlate with increased cytotoxicity of oAds and that the sequence deletion in the CR2 domain of E1A does not confer tumor specificity [136].

Therefore, continuing forward, it feels prudent to continue to study mechanisms of the brain’s biological interactions with the body and understand how the brain’s specialized immune system interacts with systemic immunity, especially in the context of its response to viral infection. This can be the lens through which to gain insight into what parts of this system can be leveraged in combination therapies. Studies optimizing delivery of either drugs or OVs can also be expanded with these more fundamental findings, as currently, most studies rely on localized intra-tumoral administration. While this is the most direct application of viruses, it is not always the most feasible when considering factors such as procedure invasiveness or the possibility of multi-dose administration. Furthermore, a combination of all this knowledge can help determine better viral modifications for better tumor and tissue targeting.

## 8. Conclusions

In this review, we have summarized recent findings regarding the application of oncolytic viruses in brain cancers as a method of immunomodulatory therapy. Namely, we have focused on the work around three more veteran viruses–HSV-1, Ad, and poliovirus–and explored other neurotropic viral candidates.

Each virus, and the strategies that aim to leverage its immunostimulatory consequences, has the following three purposes: to increase tumor specificity/sparing of healthy tissue, tumor cytotoxicity, or attenuation/safety profiling. On the whole, current pre-clinical findings seem to have explored the possibilities of the first two goals, while clinical trials have primarily demonstrated tolerable safety profiles of this treatment modality, with future proposed trials to better dissect efficacy and outcomes. However, much still remains unknown regarding unexplored combination therapies, delivery methodologies, and our understanding of the physiological mechanisms of CNS immunology in the context of cancer and viral infection. While there are many current challenges in studying the brain and its elusive contributions to the tumor microenvironment in brain tumors, such as its particular pattern of compartmentalization, lack of appropriate models, and non-conventional biology, this pathology affects one of the most impactful aspects of a patient’s self and the ways a patient understands themselves.

To conclude, the field of OVs explores another tool in the arsenal against cancer, and while much work remains to be performed, specifically in the line of brain cancer, which has always demonstrated a sort of unconventionality in both its biology and clinical presentation, it remains hopeful that each study brings the field closer to fully understanding and treating this devastating disease.

## Figures and Tables

**Table 1:** A list of ongoing oncolytic virus trials in gliomas. (Search terms: Glioma, brain tumor, oncolytic, brain cancer, recurrent, malignant, tumor).

Oncolytic Virus Clinical Trials in Gliomas					
Oncolytic Virus	Viral Backbone	Participants	Trial Status	Conditions Treated	NCT Number
HSV-1					
C134	HSV-1	12	Phase I	Recurrent glioblastoma	NCT06193174
C134	HSV-1	19	Phase I	Recurrent glioblastoma	NCT03655776
HSV G207	HSV-1	24	Phase I	Cerebellar brain tumors	NCT03911388
HSV G207	HSV-1	25	Phase I	Supratentorial brain tumors	NCT02457845
HSV-1716	HSV-1	9	Phase I	Pediatric high-grade glioma	NCT01864759
M032-HSV-1	HSV-1	29	Phase I	Malignant glioma	NCT02062827
MVR-C5252	HSV-1	51	Phase I	High-grade glioma	NCT06126744
MVR-C5252	HSV-1	51	Phase I	High-grade glioma	NCT05095441
ON-01	HSV-1	38	Phase I	Malignant glioma	NCT05626281
TG6002/5-FC	HSV-1	78	Phase I	Recurrent glioblastoma	NCT03294436
rQNestin34.5v.2	HSV-1	62	Phase I	Malignant glioma	NCT03152318
G207	HSV-1	65	Phase Ib/II	Brain cancer	NCT00028158
HSV G207	HSV-1	24	Phase II	High-grade glioma	NCT04482933
Adenovirus					
NRG-103	Adenovirus	15	Early Phase I	Glioblastoma	NCT06757153
Ad-TD-nsIL12	Adenovirus	18	Phase I	Primary diffuse intrinsic pontine glioma	NCT05717172
Ad-TD-nsIL 12	Adenovirus	18	Phase I	Progressive diffuse intrinsic pontine glioma	NCT05717899
L-IFN	Adenovirus	6	Phase I	Recurrent glioblastoma	NCT05914935
NSC-based virotherapy	Adenovirus	18	Phase I	Malignant glioma	NCT03072134
Adenovirus + Pembrolizumab	Adenovirus	49	Phase II	Malignant glioma	NCT02798406
DNX-2401	Adenovirus Δ24-RGD	12	Phase I	Diffuse intrinsic pontine glioma	NCT03178032
DNX-2401 + IFN-y	Adenovirus Δ24-RGD	13	Phase I	Glioblastoma/ gliosarcoma	NCT02197169
DNX2401 + Temozolomide	Adenovirus Δ24-RGD	24	Phase I	Recurrent glioblastoma	NCT01956734
MSC-DNX-2401	Adenovirus Δ24-RGD	37	Phase I	High-grade glioma	NCT03896568
NSC-CRAd-S-pk7	Adenovirus (conditionally replicative)	36	Phase I	Glioblastoma	NCT05130956
TS-2021	Adenovirus (Ad5 engineered)	30	Phase I	Glioblastoma multiforme	NCT06585257
BioTTT001	Adenovirus (nsIL12-expressing)	30	Phase Ib	Recurrent/progressive high-grade glioma	NCT06763965
Poliovirus					
PVSRIPO	Poliovirus	18	Phase Ib	Malignant glioma	NCT02986178
PVSRIPO	Poliovirus- rhinovirus chimera	61	Phase II	Recurrent glioblastoma multiforme	NCT01301430
Lerapolturev	Poliovirus- rhinovirus chimera	121	Phase II	Pediatric glioma	NCT03043391
Other viral backbones					
GC001	Vaccinia virus (MVA-based)	35	Phase I	High-grade glioma	NCT06660650
Parvovirus H-1 (ParvOryx)	Parvovirus H-1	18	Phase I/Iia	Malignant glioma	NCT00528684
REOLYSINR	Reovirus	18	Phase I/II	Malignant glioma	NCT00028168

Abbreviations: HSV-1, herpes simplex virus type 1; MVA, modified vaccinia Ankara; IFN, interferon; CRAD, conditionally replicative adenovirus; RGD, Arg-Gly-Asp; nsIL12, non-secreting interleukin-12.
